# Multiplex basophil activation tests for allergy diagnosis: present and future applications

**DOI:** 10.3389/falgy.2024.1515843

**Published:** 2025-01-14

**Authors:** Ana Koren, Peter Korosec

**Affiliations:** ^1^ Laboratory for Clinical Immunology and Molecular Genetics, University Clinic of Respiratory and Allergic Diseases Golnik, Golnik, Slovenia; ^2^ Faculty of Pharmacy, University of Ljubljana, Ljubljana, Slovenia

**Keywords:** allergy, diagnosis, basophil activation test, CD63, multiplex analysis

## Abstract

The basophil activation test (BAT) has become a major cellular *in vitro* test for evaluating the allergenic activity of specific IgEs. The impact of the BAT is due to the ability of blood basophil granulocytes to present IgE on the high-affinity Fc*ε*RI receptor and to mirror the mast cell response that elicits an acute allergic reaction. The BAT proved to be able to identify allergic patients at risk of reacting to a low dose of the allergen and/or developing life-threatening reactions and thus can significantly improve the current management of allergic patients. However, to improve the diagnostic utility for identifying the allergenic activity of different genuinely sensitizing allergens and lower the procedure and labour requirements, developing a multiplex BAT approach incorporating multiple allergen components would be highly anticipated. Recently, the novel multiplex BAT was described utilizing two major innovative steps. The first step was the fluorescent labeling of allergens. The second step was applying fluorescently labeled allergens in flow cytometry assessment to analyze the activation of basophil subpopulations gated according to the binding of different allergens or to evaluate the fluorescence intensity of multiple allergens on the surface of basophils. These novel cellular multiplex approaches will advance our understanding of IgE-mediated responses. Integration of multiplex BAT, in addition to multiplex IgE assays into practice, will personalize the measurement of allergenic IgE activity and can help estimate the likelihood of clinical relevance and risks for multiple allergens when testing individual allergic patients.

## 1 Introduction

The *in vitro* diagnosis of allergic diseases is based on the measurement of IgE antibodies to allergens, and the confirmation of the presence and concentration of specific IgEs in the serum determines IgE sensitization to a given allergen. This assessment was significantly advanced with the characterization and/or cloning of defined allergen molecules with established overall biologic and disease relevance; these molecules are now used for component-resolved IgE diagnostics with purified native or recombinant allergens (1, 2). Another major step was the development of simultaneous measurement of IgEs to multiple allergen components by microarray technology (1, 2). However, this highly effective and precise approach to evaluate IgE sensitization to allergen molecules cannot reveal the actual allergenic activity of the measured IgE antibodies and, thus, the ability of those antibodies to induce degranulation after IgE cross-linking with bi- or multivalent allergens. In particular, birth cohort studies show that although up to 60% of the population exhibits IgE sensitization to allergens, many individuals do not develop symptoms (1). Thus, IgE sensitization is often asymptomatic, and a recent report suggests that sialylation of IgEs might be an important determinant of these phenomena (3). Furthermore, allergic diseases range from local and mild to systemic and severe allergic reactions. Therefore, the establishment of the actual allergenic activity of a specific IgE is an important addition to the assessment of IgE sensitization, and in the past decade, the basophil activation test (BAT) has become a major *in vitro* test (4) for evaluating the allergenic activity of specific IgEs in a given subject. The impact of BAT is due to the ability of blood basophil granulocytes to present IgE on the high-affinity Fc*ε*RI receptor and to mirror the mast cell (MC) response that elicits an acute allergic reaction. Specifically, basophils become sensitized 3 h after the introduction of IgE by plasma transfusion and persist in circulation, with T1/2 ranging from 4 to 10 days (5). Blood IgE has a T1/2 of 2 days (5). The free IgE concentration determines the density of Fc*ε*RI-IgE complexes of basophils and MCs (6). Basophils have thus sampled the recent IgE distribution in the circulation, where MCs take 3 months to adapt to a change in free circulating IgE (6). MCs and basophils from a given individual will thus present similar, but not necessarily identical, repertoires of IgE.

## 2 Methodological developments of the basophil activation test

The BAT has become a major test for allergic responses through the development of flow cytometry and the discovery of activation markers and unique markers identifying basophil granulocytes (4). The main methodological milestones of the BAT are summarized in Table 1. Crosslinking of Fc*ε*RI-bound IgE by bi- or multi-valent allergens results in the activation of basophils, and two different activation markers are preferably used to assess basophil activation in the BAT: CD63, which is associated with histamine-containing granules (7), and CD203c (35), an ectonucleotide pyrophosphatase/phosphodiesterase that regulates ATP hydrolysis on the surface of basophils (10). CD63 expression is related to anaphylactic degranulation in basophils and mast cells (7, 9), while CD203c is related to piecemeal degranulation processes in basophils (9). Surface expression of CD63 and CD203c could be very accurately measured by flow cytometry (4). Basophils can be selected through several surface markers, such as high-affinity IgE receptor (FcɛRI) or IgE (11, 12), CD123+/HLA-DR- (13, 14), CCR3(CD193) (15) or CD203 (9, 16). The blood is stimulated with different concentrations of the allergen (most often logarithmic dilutions). In the positive control assay, we use the anti-Fc*ε*RI antibody and the fMLP control. A stimulation buffer without added allergen is used as a negative control (7, 11, 12, 17). The negative control is crucial to determine basal basophil activation. Allergen source is an important factor in BAT applications, ranging from allergen extracts to pure allergen molecules. Standardized allergen preparations are needed to compare the BAT results from different laboratories or performing tests over time (4). Recombinant allergen molecules are of limited availability but can improve the diagnostic performance of the BAT in some cases and have the greatest composition and stability compared to allergen extracts (2, 16, 18–20). Flow cytometric analysis offers two approaches: manual operation, which demands consistent standardization across different systems, and an automated approach (21, 22). Implementing automation in flow cytometry data analysis can increase the effectiveness and clarity of analyses and boost the outcomes' consistency (22). The BAT results can be referred to in terms of reactivity and sensitivity. Basophil reactivity refers to the number of basophils that respond at the given allergen concentration and is expressed as the percentage of basophils expressing activation marker (e.g., %CD63) (23, 24). Basophil sensitivity (CD-sens or EC50) refers to the allergen concentration at which half of all reactive basophils respond (25–29). The same concept used for the BAT, including activation markers, *in vitro* allergen stimulation, and flow cytometry analysis, was more recently used for the development of a novel mast cell activation test (MAT), which might be useful to explore differences in effector cell function between basophils and MCs during allergic reactions (30, 31).

**Table 1:** Major methodological developments of the basophil activation test.

Development	Description	References
Activation markers		
CD63	Membrane protein localized to the same secretory granule that contains histamine. Translocation of CD63 to the cell membrane during anaphylactic degranulation of basophils and mast cells	(7)
CD203c	Ectonucleotide pyrophosphatase/phosphodiesterase, which regulates ATP hydrolysis on the surface of basophils	(8) (9) (10)
Markers for identifying basophils		
IgE and FcƐRI	Varies with the plasma concentration of IgE, leading to weak staining at low IgE levels and difficulties in basophil gating	(11) (12)
CD123+ HLA-DR-	CD123 is also expressed on HLA-DR + plasmacytoid dendritic cells	(13) (14)
CCR3	Receptor for eotaxin (CD193)	(15)
CD203c	Expression increases upon manipulation of cells or during non-degranulating stimulation of basophils	(16) (9)
Stimulation control		
Stimulation buffer	Negative control	(11) (12)
Anti-IgE and anti-FcƐRI	IgE-mediated positive controls. Basophils of non-responders do not become activated upon stimulation through IgE/FcƐRI	(11) (12)
fMLP	Bacterial peptide fMLP; often used as a non-IgE-mediated positive control	(7) (17)
Allergen type		
Allergen extract	Importance of using a standardized allergen preparation	(4)
Allergen molecule	Recombinant allergen preparations or purified allergens at exact molar concentrations	(18) (2) (19) (16) (20)
Flow cytometric analysis		
Single hand-on	Standardization between systems	(21)
Automated	Data-driven flow cytometric platform for the analysis of clinical basophil activation testing	(22)
Interpretation of results		
Basophil reactivity	The number of basophils that respond to a given stimulus. Expressed as% CD63 + basophils at a given allergen concentration or as the ratio of% CD63 + to the allergen and the IgE-mediated positive control	(23) (24)
Basophil sensitivity	The allergen concentration at which half of all reactive basophils respond. Expressed as EC50 or CD-sens (1/EC50 × 100)	(25–28) (29)
Mast cell activation test		
Primary human blood-derived mast cells	Passive sensitization of human blood-derived mast cells with patient`s sera, stimulation with peanut allergen, and measurement of CD63 activation	(30)
LAD2 cells	Passive sensitization of human mast cell line LAD2 with patient plasma, stimulation with peanut allergen, and measuring of CD63 activation	(31)
Hoxb8 mast cells	Hoxb8 mast cell assay is useful for monitoring total IgE levels, culprit allergen identification and immunotherapy monitoring	(32)
Multiplex BAT and MAT		
Fluorescently labelled allergen	Fluorescence labelling of allergens with quantum dot (Qdot) nanocrystals through chemical modification	(33)
	Genetically engineered biotinylated allergen tetramerized with streptavidin-fluorophore conjugate	(34)
Fluorescent cell barcoding	Human blood-derived mast cells	(30)
	Hoxb8 mast cells	(32)
Multiparametric analysis	Cell surface staining with differently labelled allergens to gate on different basophil subpopulations with multiple activation analysis according to selected allergens	(33)
	Staining intensities of several labelled allergens on the surface of basophils	(34)
	Individually barcoded cells are pooled for acquisition, and then deconvolution analysis is performed to identify the activation status of each cell population	(30) (32)

The BAT proved to identify allergic patients at risk of reacting to a low dose of the allergen and/or developing life-threatening reactions (36) and thus can significantly improve the current management of allergic patients. However, to improve the diagnostic utility for identifying the allergenic activity of different genuinely sensitizing allergens and lower the procedure and labor requirements, developing a multiplex BAT approach incorporating multiple allergen components would be highly anticipated. In recent years, two multiplex BAT approaches have been developed (33, 34). The first step was the fluorescent labeling of allergens to directly stain allergen-specific IgE on blood basophils (33, 34). The second step was utilizing fluorescently labeled allergens in the development of a multiplex BAT approach by using cell surface staining with differently labeled allergens to gate on different basophil subpopulations, with multiple activation analyses according to selected allergens (33) or evaluation of the staining intensity of multiple fluorescent allergen tetramers through binding to IgE on the surface of basophils (34).

## 3 Allergen fluorescent labelling

### 3.1 Structure of allergens and labeling approaches

The broadest definition of an allergen is any molecule that binds IgE antibodies. The purification of allergens started in the 1960s with ragweed and grass pollen (37). Most allergens identified to date are water-soluble proteins with a molecular weight between 5 and 50 kDa. Examples of protein allergens are Bet v 1, Der p 2, Ara h 2, and Ves v 5. To date, hundreds of protein allergens from various sources have been purified, sequenced, and crystallized (www.allergen.org). Examples of nonprotein allergens are drugs, such as beta-lactam antibiotics, chlorhexidine, neuromuscular relaxants, and analgesics. Our understanding of the allergenicity of these compounds relies on the hapten concept of a strong (covalent) bond with a carrier protein (38). Another explanation might be metabolites are an allergologically active substance. Other important nonprotein allergens are glycans, allergenic as glycoproteins or even glycolipids. Prototypic glycans with well-established IgE-binding activity are cross-reactive carbohydrate determinants (CCD) (39) and the alpha-Gal epitope (40). Glycans are produced in various plants and are also found in pollen and *Hymenoptera* venom. On the other hand, alpha-Gal is produced by nonprimate mammals, while IgE antibodies against alpha-Gal in humans most likely occur in response to tick bites (1).

**Figure 1:**
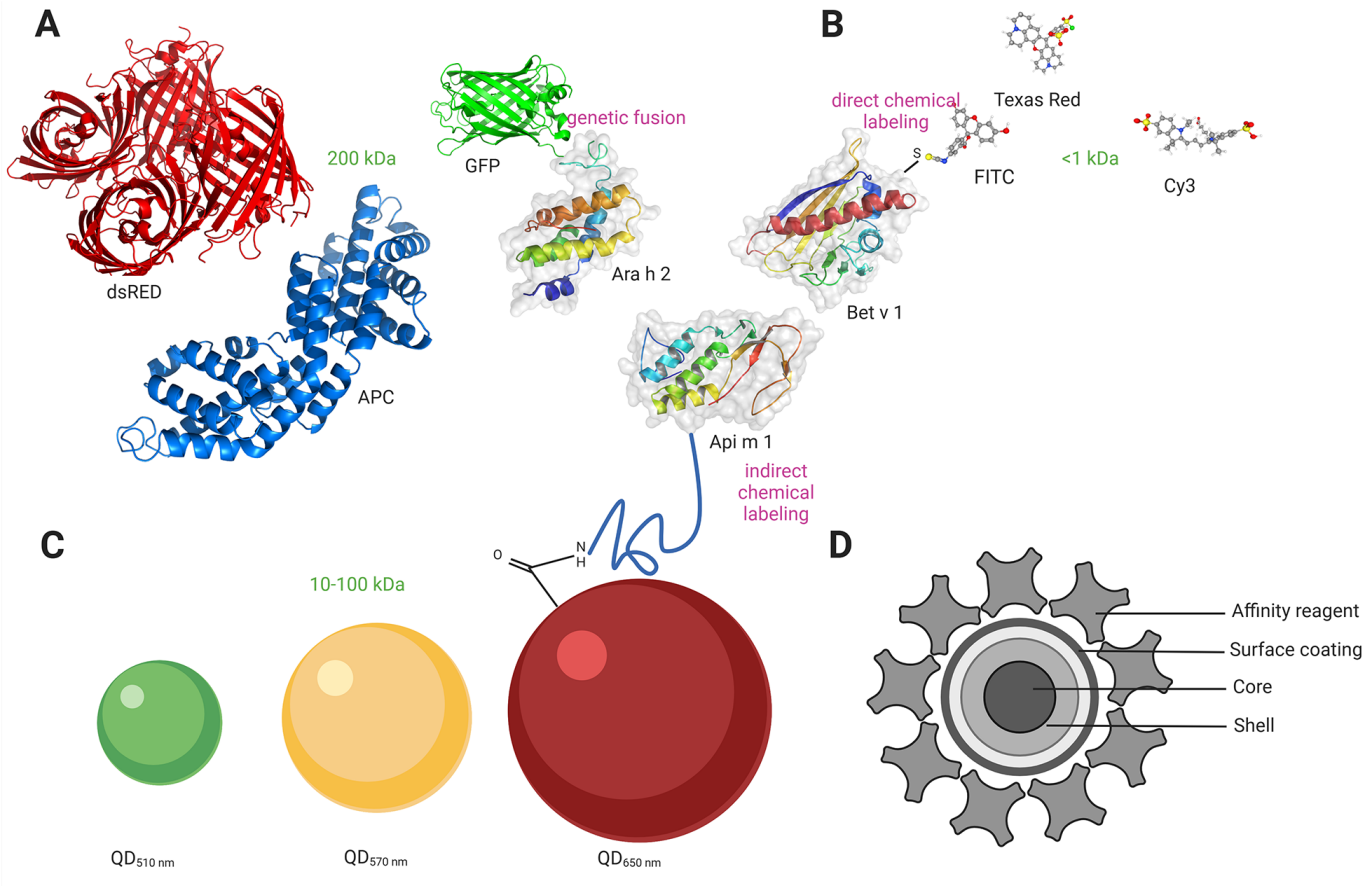
Allergen labeling with different fluorophores. **(A)** Labeling with genetically encoded fluorescent proteins (size range 200 kDa). GFP is fused to the amino terminus of the major peanut allergen Ara h 2 directly via the carboxyl terminus of approximately ten amino acids. **(B)** Labelling with small organic fluorophores (size range <1 kDa). FITC is conjugated through a free amino group on the major birch allergen Bet v 1, forming a stable thiourea bond. **(C)** Labelling Qdot nanocrystals (size range 10-100 kDa). Qdots (with an emission maximum at 650 nm) with an amino (PEG)-functionalized polymer coating are conjugated to the major honeybee venom allergen Api m 1. **(C)** Structure of Qdot nanocrystals comprising core, shell, and surface coating. An affinity reagent is bound to the polymer coat, which allows the binding of Qdots to other biomolecules. Created in https://BioRender.com. GFP, green fluorescent protein; dsRED, red fluorescent protein; APC, allophycocyanin; FITC, fluorescein isothiocyanate; Qdot, quantum dot; PEG, polyethylene glycol; Ara h 2, Ara h 2 peanut; Api m 1, Api m 1 Phospholipase A2, honeybee; Bet v 1, Bet v 1 PR-10, Birch.

Ideally, a fluorescent label should be small, bright, and stable and should not interfere with the functions of the biological system. Additionally, labels should be specific for the target molecule, preferably establishing a covalent linkage between the fluorescent probe and a specific residue on the target protein. It should also not label multiple proteins and, therefore, form oligomers. Reliable allergen labeling requires a proficient understanding of protein chemistry. There are three main groups of existing fluorescent labels: fluorescent proteins, small organic dyes, and inorganic crystals (Figure 1). The important features to consider when choosing the best fluorescent label for a specific application are fluorophore brightness, fluorescence lifetime, fluorophore size, and photostability (41). Fluorescent molecule attachment to the allergen can be achieved biologically (genetic incorporation of unnatural amino acids) or chemically (chemical modification through noncovalent or covalent binding). In addition to the abovementioned biological and chemical labeling methods, a new type of labeling is widely employed (i.e., tag labeling), which can be carried out both chemically and biologically (42).

### 3.2 Fluorescent proteins

Fluorescent proteins can be genetically engineered to produce fusion proteins with the target protein/allergen or attached through chemical/biological modification of the target protein. Genetically encoded fluorescent proteins are protein sequences that can be fused by recombinant cloning to a protein of interest at the N- or C-terminus to render fluorescent (43–45). The first fluorescent protein used as a fluorescent marker was green fluorescent protein (GFP) from *Aequorea Victoria* in the 1990s (46, 47). eGFP (enhanced GFP), YFP (yellow fluorescent protein), and RFP (red fluorescent protein) are GFP variants that show better brightness and increased photostability (48). To date, several fluorescent proteins have been developed, providing additional wavelengths, increased stability, higher brightness, and photoactivation properties (44, 49, 50). The advantage of the genetic labelling approach is that it is most specific since there is no background signal from the free fluorophore. This is also the easiest method to use for live cell measurements. The second group of fluorescent proteins is those derived from the phycobiliproteins found in algae and plants. These proteins use phycobilin cofactors to harvest light and include phycoerythrin (PE), allophycocyanin (APC), and peridinin chlorophyll (PerCP) (Figure 1A). Due to their large size (200 kDa), their application is mainly in antibody conjugates for surface labeling in flow cytometry (51). Chemical covalent attachment of the fluorescent protein to the target protein can be achieved by modifying amine or cysteine groups. There are some important points to consider when creating a functional fluorescent protein: the fluorescent protein must fold correctly to fluoresce, the host protein must also fold correctly to be functional, and the integrity of the chimeric protein must be maintained. The linker between the fluorescent protein and the target protein should be sufficiently long and flexible to prevent steric hindrance or folding interference. A common way to link two protein domains is through several glycine residues (52). Additionally, fluorescent proteins are very large labels (30 kDa/4 nm), and some, such as pZsGreen, dsRed, or GFP, tend to oligomerize into tetramers (53). More recently, mutant variants have been developed to reduce this effect (44, 53).

### 3.3 Small organic fluorophores

Fluorescent dyes are small (<1 kDa), organic natural or synthetic fluorescent molecules. Some of the fluorophores in this group include fluorescein isothiocyanate (FITC), sulforhodamine 101 acid chloride (Texas Red), and cyanine (Cy) (Figure 1B). An important benefit of using small organic fluorophores is minimizing possible steric hindrance problems that can interfere with protein function. Other favorable features are a wide spectral range and, in some cases, high brightness, making them the most exploited probes in cell biology. However, traditional fluorophores have limitations. Irreversible light-induced changes can occur after light excitation, resulting in a phenomenon known as photobleaching. Another limitation is broad overlapping emission lines, significantly affecting multicolor detection.

Coupling of the label to a target protein can be achieved by direct and indirect labeling methods. Direct chemical labeling of proteins targets cysteine and amine groups. The greatest problem is controlling the specificity of the labeling. Cysteines provide the greatest flexibility for choosing the labeling location due to their low frequency. Since labeling is not always 100%, the free dye may result in high background and nonspecific signals. Therefore, it is important to distinguish the labeled from the unlabeled protein. Purification of the labeled and non-labeled protein can be achieved with ion exchange or size exclusion chromatography. Indirect labeling techniques include peptide or protein tags for organic fluorophores. Peptide tagging can be performed by two main approaches: labeling a synthetic peptide that is then attached to the target protein through ligation or genetic engineering of peptide tags onto the target protein, which is then reacted with the corresponding fluorophore conjugates. Protein tags such as Halo (54), SNAP (55), and CLIP (56) are self-labeling enzymes that covalently link to fluorescently labeled substrates.

### 3.4 Quantum dot nanocrystals

Quantum dot (Qdot) nanocrystals are protein-sized (10–20 nm) atom clusters comprising a core, shell, and surface coating. The core is made of semiconductor material [cadmium selenide (CdSe) or cadmium telluride (CdTe)]. A semiconductor shell [typically zinc sulfide (ZnS)] surrounds and stabilizes the core, improving the optical and physical properties of the material (57). The core-shell assembly is strongly hydrophobic; therefore, an amphiphilic polymer coating is then applied, serving two purposes. First, it incorporates ionizable functional groups that confer water solubility essential for bioanalytical applications, and second, it provides a platform for covalent functionalization with antibodies, streptavidin, or other affinity reagents. Qdot nanocrystal conjugates typically incorporate multiple copies of the affinity reagent, contrary to the organic dye-labeled conjugates in which multiple dyes are attached to a single affinity reagent. The affinity reagent can be coupled to the amphiphilic polymer coating via a functionalized polyethylene glycol (PEG) linker, which has been shown to reduce steric hindrance and nonspecific binding by flow cytometry (Figures 1C,D).

Qdots have unique optical properties and provide several important advantages over fluorescent proteins and small organic dyes, such as narrow and symmetric emission spectra and high fluorescence and photostability, making Qdots promising in several biological applications. The Qdot emission wavelength can be quickly and precisely tuned by adjusting the nanocrystal size; consequently, multicolor nanocrystals of different sizes can be excited by a single light source with minimum signal overlap. Importantly, acquisition samples using Qdots often exhibit better brightness due to their high resistance to bleaching, which is one of their most important advantages. This is due to the lack of excitation-induced damage and lower exposure of the fluorescence center to the solvent. Qdots are compatible with other existing organic dyes, making them easy to incorporate into existing experimental setups. An important feature to consider is the large size of Qdots, which may interfere with the activity of the target protein; therefore, proper control experiments should be performed.

## 4 IgE-binding and allergenic activity of labeled allergens

An immediate allergic reaction occurs through allergen cross-linking of specific IgE antibodies, which are bound to effector cells (mast cells and basophils) through high-affinity IgE receptors and consequent degranulation and release of inflammatory mediators (2, 58). IgE antibodies of allergic patients are usually directed against conformational epitopes on properly folded allergens or allergen domains (59–61). Cross-linking of IgE antibodies requires at least two IgE epitopes on an allergen molecule (bivalent) to activate effector cells (62–65). The potency of an allergen depends on factors determined by the IgE-binding epitopes of an allergen. It increases with the number of IgE epitopes on the allergen (66), the proximity of the epitopes (67), and the affinity and levels of allergen-specific IgE (68, 69). Allergenicity also increases when epitopes are present on two allergen domains that are connected by flexible linkers that allow optimal engagement of effector cell-bound IgEs (70). Conversely, allergen aggregates display unfavorable bivalent IgE-binding sites and thus exhibit reduced allergenic activity, although IgE reactivity is preserved (71, 72). Fluorescence labeling may affect the allergen's capacities to bind and/or cross-link IgEs through a reduction in interaction sites between paratopes on IgE and epitopes on the allergen. This might be particularly important in the case of allergens with a small number of epitopes or when epitopes are in an unfavorable distance and/or position (71, 72). This reduction might be induced by chemical or physical interactions with fluorescent dyes. In that case, the allergen epitopes might become modified and/or physically covered in a way that they are no longer able to bind the IgEs. For example, if fluorescent labels are large (t.i. fluorescent protein, Qdot), they can spherically (physically) interfere with the allergen and prevent contact between the epitope and cell-bound IgEs. Specifically, we recently demonstrated that even if carboxyl-conjugated Qdots (with carboxylate-functionalized coatings) provide more binding sites for allergens, such Qdot-labelled allergens retain only IgE reactivity (i.e., monovalent binding capacity), but they lose IgE allergenic and crosslinking activity (i.e., bivalent binding capacity). In contrast, amino-conjugated Qdots (with amino-functionalized coatings) contain a flexible PEG linker, which can distance allergens away from the Qdot, and such Qdot-labelled allergens retain both monovalent IgE binding capacity and bivalent IgE allergenic and crosslinking activity (33). Thus, we might speculate that better access to the epitopes in the case of labelling allergens with amino-conjugated Qdots and flexible PEG linkers is the major cause for those significant differences. Therefore, we might consider allergen-fluorescent conjugates as multimeric allergens with different availability of epitopes for IgE binding. Consequently, their allergenic properties might be slightly different compared to the unlabelled allergen. Interestingly, in a recent report, Qdot-labelled allergens induced shifts between basophil response curves and/or maximal CD63 response or lower sensitivity of the BAT in comparison to native allergens in patients with low levels of sIgEs but not in patients with higher levels of sIgEs (33).

## 5 Multiplex basophil activation test analysis

The first multiplex approach in allergology dates back to 2002, with the development of IgE microarray technology, which allowed the simultaneous testing of IgE reactivity for more than 100 different allergen molecules (73). Various allergen components were spotted on a polymer-coated glass slide in this approach. After incubation with the patient's sera, allergen-bound IgE antibodies were detected using labeled anti-human IgE, and fluorescence was measured with a laser scanner (73). The major clinical and laboratory advantages of the multiplex approach are the resolution of complex sensitization profiles of multisensitized patients and the identification of genuinely sensitizing allergens (74). Applying allergen components to the BAT was another step that further improved the diagnostic accuracy of *in vitro* testing (19, 75).

**Figure 2:**
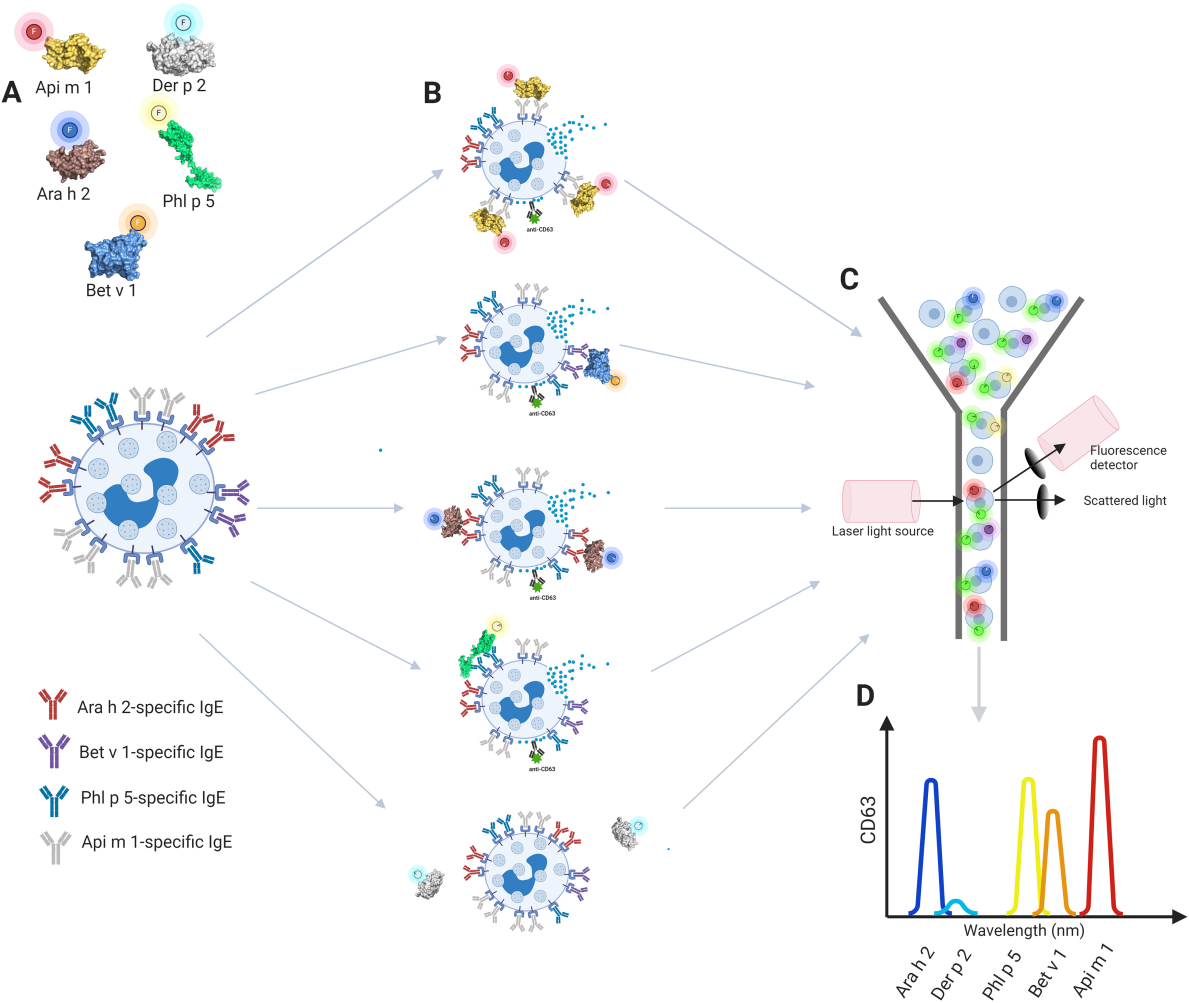
The concept of the multiplex basophil activation test. **(A)** Fresh whole blood basophils are separately stimulated with allergens labeled with different fluorophores, **(B)** aliquots are pooled together for CD63 activation staining and **(C)** flow cytometry assessment is performed as a single sample. **(D)** Gating of different basophil subpopulations with multiple activation analysis according to selected allergens. Created in https://BioRender.com. Ara h 2, Ara h 2 Peanut; Bet v 1, Bet v 1 PR, 10 Birch; Phl p 5, Phl p 5 Timothy; Api m 1, Api m 1, Phospholipase A2, honey bee.

With the introduction of fluorescently labeled allergens (33, 34), it has recently become feasible to develop a multiplex BAT in which we can use differently labeled allergens to simultaneously analyze the activation of basophil subpopulations gated according to the binding of specifically labeled allergens (33) or to evaluate the fluorescence intensity of multiple allergens on the surface of basophils (34). Both methodologies exploit the concept that basophils present high levels of IgE on their surface through FcɛRI binding and that this IgE is polyclonal. In the first approach (33), fresh whole blood was separately stimulated with different (logarithmic) concentrations of labeled allergens (Figure 2A), and then aliquots were pooled together for basophil marker and activation staining and flow cytometry assessment as a single sample (Figures 2B,C). Importantly, individual stimulation in the first step was needed to pinpoint degranulation levels for every specific allergen, and activation (quantitative surface expression of CD63) was then analyzed simultaneously for different subpopulations of basophils according to the binding of the fluorescent allergens (Figure 2D). This allows simultaneous activation and analysis of the cluster of allergens, rather than running a single assay for each allergen tested as in a conventional BAT. In the second approach (34), termed CytoBas, fresh whole blood or alternatively fresh frozen peripheral blood mononuclear cells were stained with fluorescently labeled recombinant allergens and basophil markers in one step, and then the fluorescence intensity of the allergen on the surface of basophils was measured with flow cytometry. CytoBas is an interesting alternative to serology-based component-resolved IgE testing with additional value, providing insight into the distribution of IgEs on the surface of basophils. However, this direct single-step approach, without the *in vitro* stimulation step, does not provide any information regarding the allergenic activity of IgEs on the surface of basophils. Overall, according to economical and practical points, both approaches significantly reduce the use of consumables, reagents, equipment operation, cells (fewer samples for acquisition), and analysis time compared to the conventional BAT.

Fluorescent cell barcoding (FCB) may represent a further improvement of multiplex BAT, enabling even higher throughput (76). In the FCB approach, individual cell samples are barcoded with unique signatures of fluorescent dyes so that they can be pooled together, stained, and analyzed as a single sample (76). Current clinical flow cytometers allow the development of multiplex BAT with 10 or more allergens, with the potential to use complex multicolor analysis with up to 50 simultaneous parameters. With that in mind, it will be important to further standardize the flow cytometer instrument settings and include appropriate controls for reproducible and comparable BAT results (77, 78). The technology of allergen fluorescence labeling and FCB could also be translated to MAT (30, 32), which would enable the interpretation of IgE allergenic activity in patients with nonresponding basophils (4, 79) and facilitate the procedure in cases when fresh blood for the BAT cannot be obtained.

## 6 Discussion

To integrate multiplex BAT analysis into diagnostics, it is essential to first perform detailed comparisons between labeled and unlabeled allergens for each allergen in the BAT setting. This step is necessary because fluorescently labeled allergens are considered multimeric, exhibiting properties distinct from pure allergens. Consequently, their binding and activation characteristics may not be entirely comparable. Our experience shows that this difference can lead to shifts in dose-response curves and/or maximal CD63 responses. Nonetheless, available data suggest that singleplex and multiplex BAT exhibit comparable sensitivity and specificity (33). The complexity of multiplex BAT is significantly higher than singleplex BAT due to the additional step of allergen labeling. Therefore, allergen labeling must be highly specific to preserve the allergen's native allergenic activity.

The multiplex BAT approach is suitable for all protein allergens, including those from food, insect venom, inhalants, and biologicals. However, special consideration is required for drug allergens, typically small molecules that may only become allergenic when covalently bound to a carrier protein. Multiplex BAT is also effective for analyzing cross-reactive allergens, following procedures similar to singleplex BAT. In such cases, the level of basophil activation depends on which cross-reactive allergen is tested. For successful fluorescent labeling, it is critical to use allergens of high purity. As a result, this technique is unsuitable for labeling whole allergen extracts; instead, it can be applied to individual allergen components that have been purified natively or produced through recombinant methods. The presence of allergen-specific blocking IgG4 antibodies can influence multiplex BAT results, similar to their effect on singleplex BAT. These antibodies bind to allergen epitopes, blocking interactions with IgE on cells. Since fluorescent labeling must preserve epitope integrity, it is expected that antigen-specific IgG4 antibodies will similarly affect multiplex BAT outcomes.

The potential clinical utility of multiplex BAT *in Hymenoptera* venom allergy lies in identifying the culprit venom for immunotherapy in patients who are serologically double-sensitized to bee and wasp venoms. A recent study (20) demonstrated that BAT using single components can aid in selecting clinically relevant venom for immunotherapy in cases with inconclusive results. With multiplex BAT, a comprehensive panel could be designed to test such patients for various allergen-specific and cross-reactive components of bee and wasp venoms. In food allergy diagnostics, BAT may help reduce the need for oral food challenges, particularly in cases with inconclusive skin prick and specific IgE (sIgE) test results (80). Multiplex BAT could serve as a panel for detecting key food storage proteins associated with severe food anaphylaxis, thus enhancing diagnostic precision. Further, multiplex BAT could also serve as a panel for detecting key respiratory allergens (pollen, animal, mold, or mite) associated with asthma or allergic rhinitis.

Current studies on multiplex BAT have demonstrated a strong correlation with singleplex BAT in detecting clinically relevant basophil activation and sensitization (33, 34). This is likely due to the specific allergen labeling techniques used (34) and the preservation of allergenic activity during the process (33). However, as multiplex BAT evolves to include a significantly higher number of allergens, maintaining concordance with singleplex BAT results may become challenging. Modern flow cytometers can simultaneously analyze up to 50 parameters, representing the theoretical upper limit for allergen numbers in multiplex BAT. Yet, including more allergens may introduce additional challenges, such as steric hindrance between allergens, which could reduce concordance between singleplex and multiplex BAT results. These limitations may ultimately restrict the maximum number of allergens that can be analyzed simultaneously. To address such discrepancies, robust statistical methods, such as Bland-Altman analysis (81), will be essential to evaluate the agreement between multiplex and singleplex BAT results. As the number of allergens analyzed increases, artificial intelligence techniques will likely play a crucial role in managing and interpreting complex data. Cluster analysis approaches, for example, could help streamline the analysis of large datasets.

Currently, multiplex BAT is not economically feasible for routine use in clinical laboratories due to the complexity and sophistication involved in fluorescent allergen labeling. However, if labeled allergens were standardized and made commercially available, multiplex BAT could become a viable diagnostic tool, offering both economic and practical benefits.

## 7 Conclusions and future directions

The availability of appropriate methods for fluorescent labeling of allergen molecules and recent advances in multicolor flow cytometry enabled the development of novel multiplex BAT assays. Fluorescent allergen labeling enables staining of allergen-specific IgE on the basophils, which can then be analyzed with flow cytometry to assess staining intensities with various allergens and multiplex activation analysis, to analyze basophil responses to multiple allergens at the same time. An important step is the allergen fluorescent labeling, which should preserve multivalent allergenic binding, which is crucial for basophil activation. The allergenic properties of the fluorescent allergens might be different compared to the unlabelled allergens, which should be considered when determining the optimal concentration ranges of fluorescent allergens in the BAT. Different fluorophores have different properties in terms of excitation and emission spectra, which should be taken into account when designing multiplex BAT experiments. The limitation of the existing multiplex BAT approach is that stimulation with each allergen separately is needed to distinguish between basophil activation in response to different reagents. Nevertheless, a significant reduction in consumables and reagents, equipment operation and cell and data analysis is observed compared to the conventional BAT. In addition, multiplex basophil analysis offers new insight into the distribution of IgE on the surface of basophils, which will advance our understanding of IgE-mediated responses and yield further information on the allergenic activity of multiple allergens. Future integration of multiplex BAT, in addition to multiplex IgE assays, into practice will personalize the measurement of allergenic IgE activity and can help estimate the likelihood of clinical relevance and risks for multiple allergens in individual allergic patients.
